# Design and Refinement of a Data Quality Assessment Workflow for a Large Pediatric Research Network

**DOI:** 10.5334/egems.294

**Published:** 2019-08-01

**Authors:** Ritu Khare, Levon H. Utidjian, Hanieh Razzaghi, Victoria Soucek, Evanette Burrows, Daniel Eckrich, Richard Hoyt, Harris Weinstein, Matthew W. Miller, David Soler, Joshua Tucker, L. Charles Bailey

**Affiliations:** 1 The Children’s Hospital of Philadelphia, US; 2 Seattle Children’s Hospital, US; 3 Nemours Children’s Health System, US; 4 Nationwide Children’s Hospital, US

**Keywords:** CDRN, Checks, Data Quality, Electronic Health Records, GitHub, Issues

## Abstract

**Background:**

Clinical data research networks (CDRNs) aggregate electronic health record data from multiple hospitals to enable large-scale research. A critical operation toward building a CDRN is conducting continual evaluations to optimize data quality. The key challenges include determining the assessment coverage on big datasets, handling data variability over time, and facilitating communication with data teams. This study presents the evolution of a systematic workflow for data quality assessment in CDRNs.

**Implementation:**

Using a specific CDRN as use case, the workflow was iteratively developed and packaged into a toolkit. The resultant toolkit comprises 685 data quality checks to identify any data quality issues, procedures to reconciliate with a history of known issues, and a contemporary GitHub-based reporting mechanism for organized tracking.

**Results:**

During the first two years of network development, the toolkit assisted in discovering over 800 data characteristics and resolving over 1400 programming errors. Longitudinal analysis indicated that the variability in time to resolution (15day mean, 24day IQR) is due to the underlying cause of the issue, perceived importance of the domain, and the complexity of assessment.

**Conclusions:**

In the absence of a formalized data quality framework, CDRNs continue to face challenges in data management and query fulfillment. The proposed data quality toolkit was empirically validated on a particular network, and is publicly available for other networks. While the toolkit is user-friendly and effective, the usage statistics indicated that the data quality process is very time-intensive and sufficient resources should be dedicated for investigating problems and optimizing data for research.

## Background

Collaborations across multiple institutions are essential to achieve sufficient cohort sizes in clinical research and strengthen findings in a wide range of scientific studies [1,2]. Clinical data research networks (CDRNs) combine electronic health record (EHR) data from multiple hospital systems to provide integrated access for conducting large-scale research studies. The results of CDRN-based studies, however, come with the caveat that the EHR data are directed towards clinical operations rather than clinical research. Suboptimal quality of EHR data and incorrect interpretation of EHR-derived data not only lead to inaccurate study results but also increase the cost of conducting science [3]. Hence, one of the most critical aspects in building a CDRN is conducting continual quality evaluation to ensure that the patient-level clinical datasets are “fit for research use” [3,4,5]. A well-designed data quality (DQ) assessment program helps data developers in identifying programming and logic errors when deriving secondary datasets from EHRs (e.g. an incorrect mapping of patient’s race information into controlled vocabularies). Also, it assists data consumers and scientists in learning the peculiar characteristics of network data (e.g. “acute respiratory tract infections” and “attention deficit hyperactive disorder” are likely to be among the most frequent diagnoses in a pediatric data resource) as well as helps assess the readiness of network data for specific research studies [6].

This study focuses on the pediatric learning health system (PEDSnet) CDRN which provides access to pediatric observational data, drawn from eight of the nation’s largest children’s hospitals, through an underlying common data model (CDM) [7]. PEDSnet is one of the 13 CDRNs supported by the Patient-Centered Outcomes Research Institute (PCORI). PEDSnet has a centralized architecture, where patient-level datasets from various hospitals are concatenated together, with an exception of two hospitals that participate in a distributed manner. The ultimate goal of PEDSnet is to provide high-quality data for conducting a variety of pediatric research studies. Therefore, an essential task during building PEDSnet was to build a system to conduct DQ assessments on the network data. We experienced three critical demands in conducting such assessments in PEDSnet.

- Assessment coverage: It is important to ensure that the key variables in the CDM are being assessed, and that the assessments encompass the important aspects of DQ [5,8] and meet the demands of internal and external data consumers. The challenge lies in selection of variables to be assessed, and the types of assessment to be used for the select variables, given plethora of possibilities of DQ checks for a single variable or a combination of variables. In a CDRN like PEDSnet that contains hundreds of variables in the CDM and a diverse set of users, identification of appropriate assessment coverage is a vital but resource-intensive task. For example, the field *condition_concept_id* that captures the standardized SNOMED diagnosis in PEDSnet could be assessed in a number of ways such as missing or unmapped diagnosis, incorrectly mapped diagnosis, variability in frequency distribution of certain diagnosis across sites, inconsistency between diagnosis and medication data, etc.
- Evolution-friendly: PEDSnet is a continually growing network; the size of the centralized dataset increases as data for new patients and new observations get added into individual EHRs. In addition, the underlying model also evolves based on the changing needs of the data consumers. For instance, the PEDSnet patient population increased by over 90 percent since the first data submission and at least six versions of the underlying model have been adopted in the last two years. It is a challenge to conduct DQ assessments while accounting for temporal variability on such an evolving dataset.
- Communication: The PEDSnet data committee is responsible for developing network data and represents a collaboration of more than 50 programmers, analysts, and scientists spread across the eight participating institutions. It is important to enable effective communication among them and track all DQ related interactions.

While much information on DQ assessments is made available by existing data sharing networks [9], there is limited discussion on the coverage and driving factors of the assessments to be encapsulated in a toolkit. There have been recent advances in conducing DQ assessments on an evolving dataset to review temporal variations [10,11,12,13]. However, the communication challenge is largely underexplored in the context of CDRNs. In this study, we describe the design and evolution of a software toolkit that addresses the key challenges outlined above and serves as a systematic DQ assessment workflow for the PEDSnet CDRN.

## Implementation

### PEDSnet Data Quality Conceptual Schema

The DQ assessment workflow in PEDSnet is based on a data quality conceptual schema that provides a map of various data quality concepts and their relationships; for further information please refer to the additional supplemental content (Figure S1). The PEDSnet network is being developed in iterations known as *data cycles*. During each data cycle, a PEDSnet *site* conducts the extract-transform-load (ETL) operations on their source EHR to prepare an instance of the PEDSnet CDM; extracting and transforming data for various *domains* and *fields* to follow PEDSnet ETL conventions [14]. The PEDSnet CDM is an adaptation of the Observational Medical Outcomes Partnership (OMOP) CDM [15]: a widely accepted schema for observational medical data [2]. In the PEDSnet CDM, certain additional fields and domains have been added to meet pediatric research needs. For example, the fields gestational_age and time_of_birth have been added to the person table. Additional domains like visit_payer for patient insurance information and adt_occurrence to track patient location were also added to the CDM. The sites submit these CDM-aligned datasets (i.e. with a certain *etl convention version*) to the PEDSnet Data Coordinating Center (DCC) which then conducts the DQ assessments on these datasets.

In PEDSnet, a *check type* is a category of DQ assessment to be performed on the dataset. A *check* is instantiated when a check type is applied to a specific field in the CDM. The threshold attributes are associated with a check that returns a numerical value and denotes the range of acceptable values for that check. If the returned value is outside the threshold bounds, a DQ *issue* is created. A DQ issue is the conceptual result of executing a check on a site’s dataset. The data quality workflow returns the description of the issue and the corresponding GitHub link for the issue (discussed further in the “DQ Feedback and Resolution” subsection). The DCC manually updates the *status* and *cause* of the issue as the data cycle progresses. Table 1 shows examples of three DQ issues and their meta-information in PEDSnet.

**Table 1:** Examples of check type, check and data quality issue in PEDSnet.

Entity	Attribute	Example-1	Example-2	Example-3

**Check Type**	*Name*	unexpected most frequent values	Pre-birth fact	unexpected change in number of records between data cycles
	*Alias*	*UnexTop*	*PreBirth*	*UnexDiff*
**Check**	*Lower_threshold*		0	0
	*Upper_threshold*		0	15
**Field**	*Name*	Condition_concept_id	Visit_start_date, time_of_birth	
	*Data_type*	Numeric	Date, date	
**Domain**	*Name*	condition_occurrence	visit_occurrence, person	drug_exposure
**Data Quality Issue**	*Description*	Shooting pain (OMOP concept_id: 4171519)	11557 visits before patient was born	22.65%
	*Status*	Solution proposed	persistent	withdrawn
	*Cause*	ETL: programming error	Characteristic: true anomaly	False alarm: improvement in previous ETL
**Observed_at**	*Data_version*	ETLv11	ETLv8	ETLv10
**Data_cycle**	*Cycle_date*	September 2016	April 2016	September 2016
	*ETL_conventions_version*	2.4.0	2.2.0	2.4.0

At a given point in a data cycle, an issue may be in one of the five states: new, under review, solution proposed, persistent, and withdrawn. An issue is *new* when it is identified by the workflow in the current data cycle for the first time and becomes *under review* when it is being reviewed by the site; from this state the issue may proceed to one of the following three states: when there is a solution in place to resolve the issue in the next data cycle (*solution proposed*), when the issue is likely to persist in the next data cycle (*persistent*), and when the issue is a false alarm by the DCC (*withdrawn*).

### PEDSnet Data Quality Workflow

**Figure 1:**
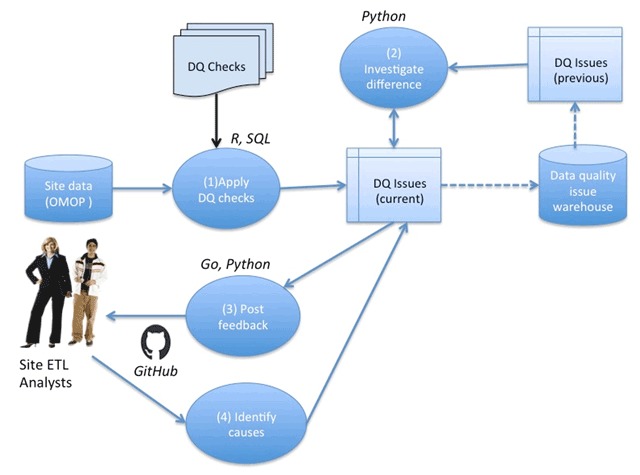
The PEDSnet data quality assessment workflow.

The workflow for conducting DQ assessments on a given PEDSnet partner site is illustrated in Figure 1. Upon receiving the site dataset, packaged in the PEDSnet CDM, the data is loaded into a database, and the integrity constraints and data model conformity are validated, then (1) a series of DQ checks are applied to identify any DQ issues associated with the submitted dataset, (2) the identified DQ issues are compared with a list of issues associated with the previous data cycle submission by the site to identify any conflicts and the current list of DQ issues is updated accordingly, (3) the DQ issues are translated into GitHub issues and posted for site review and discussion on the site-specific private repository, and (4) finally, the cause (ETL vs. characteristic vs. false alarm) of each issue is determined based on the GitHub discussions.

### DQ Check Design

The DQ checks were developed and evolved in multiple phases. During the first phase, the data quality checks were designed solely based on theoretical knowledge and literature review [2,3,5]. The checks were developed by a pediatrician and a data scientist, covering aspects such as fidelity, consistency, accuracy, and feasibility [3]. The second phase began after we started conducting data cycles in PEDSnet. At the end of each data cycle, PEDSnet data committee members (>50 members) proposed new checks and edits to existing checks (e.g. threshold modifications) based on data reviews and issue investigations. The third and current phase represents the development of DQ checks as PEDSnet started accepting science (study-specific) queries from researchers across the nation [16]. Each science query led to the discovery of new (previously undetected) data quality issues that necessitated the design of a new data quality check. In addition, at the end of each cycle, new checks are also designed based on changes in the underlying CDM and related conventions (e.g. addition of new fields or domains or value sets.)

### DQ Check Implementation

For implementing the checks, we used a variety of computational methods [5] such as:

- Data element agreement: Two or more elements within a site dataset are compared to see if they report the same or compatible information. This method is used for identifying inconsistency between date and datetime fields within a given domain (*InconDate*), inconsistency between types of visits captured in two different domains (*InconVisitType*), inconsistency in null values between *_source_value fields containing untransformed data from the EHR and *_concept_id fields containing data mapped to the OMOP standard vocabulary (*InconSource*), and identifying event start dates that occur after the event end dates (*ImplEvent*), etc.
- Element presence: This method checks for the presence of desired or expected data elements. This method is used to compute data completeness for fields (*MissData*), presence of expected value from a domain (*MissFact*), completeness of mapping of facts to standard vocabularies (*MissConceptID*), or coverage of sufficient facts for encounters (*MissVisitFact*).
- Data source agreement: The site data, as submitted to the DCC, is compared with data from another source to determine if they are in agreement. This method is used to identify whether there is an unexpected change in number of records in a given domain (*UnexDiff*), or data completeness in a given field (*UnexMiss*), between consecutive data cycles.
- Distribution comparison: The distributions of clinical concepts of interest across site data are compared with the expected distributions, as determined through internal or external sources, in order to identify any outliers. An example is the unexpected value (*UnexTop*) check that reviews the frequency distribution (or rank) of a diagnosis at a site, e.g. if a diagnosis appears as the top-ranked diagnosis at the site, and does not appear among the top 50 at other sites, it is considered an outlier and hence a data quality issue is generated. Another example is the temporal outlier (*TempOutlier*) check that reviews the longitudinal distributions of facts, such as number of visits per month, and identifies outlier months, e.g. sudden increase or decrease in number of visits, using standard statistical methods.
- Face validity check: Site data are assessed using various techniques that determine if values ‘make sense’. This method is used to ensure that the data are aligned with the PEDSnet ETL conventions, e.g. value set violations (*InvalidConID, InvalidVocab*), inclusion criteria violations (*InconCohort*), or whether the data are clinically plausible, e.g. identifying facts occurring in the future (*ImplFutureDate*), or after a patient’s death (*PostDeath*), or numerical outliers (*NumOutlier*).

### Cross-cycle Difference Investigation

A key challenge in conducting DQ assessments on an evolving CDRN is to track and question the variation of data characteristics from one cycle to another. For each identified issue *i_Curr_* in the current data cycle, the DQ warehouse is first searched to see if a “similar” issue *i_Prev_* persisted (*persistent* or *under review*) in the previous data cycle, wherein similarity is based on the associated field(s) and check type of both the issues. Then, the difference between the numerical findings of the two issues is computed. If the difference lies outside the range of pre-defined threshold bounds, a new data quality issue is created for sites to investigate the difference. Otherwise, the status of the current issue *i_Curr_* is marked as “persistent” or “under review” to align with *i_Prev_*. As an example, in PEDSnet we accept –10 percent to +10 percent variation in the missingness of values in a field. If *i_Curr_* states that 50 percent of the condition_occurrence records do not have a condition_end_date and *i_Prev_* has 30 percent missingness in condition_end_date as a persistent issue, a new issue will be created to investigate this difference, whereas if *i_Prev_* had 55 percent missingness, a new issue would not be created. The flowchart for this process is provided as an additional supplemental content (Figure S2).

### DQ Feedback and Resolution

In PEDSnet, we adopted GitHub as a tool to manage all data quality related communications [17]. Initially, the workflow was programmed to generate a single GitHub issue, enlisting all DQ issues, in the private repository for the given site. A few months into the data cycles, we adopted a more modular approach based on the “status” of issues. For each *new* DQ issue, the workflow opens a new GitHub issue in the source site’s repository. The body of the GitHub issue contains the check type, finding, and a hyperlink to the DQ workflow source code module that contains the programming logic of the underlying check type. For each *under review* issue, the workflow redirects to the existing GitHub issue that would have been opened in the previous data cycle. The issues marked as *persistent* are not further propagated. The workflow automatically assigns certain labels to various GitHub issues denoting the data cycle, status, and domain associated with the issue. The labels allow filtering and sorting through the issues. This GitHub-based collaborative system helps in automatically tracking the status of issues and documenting the interactions with each site. Based on the site’s responses, the PEDSnet DCC team edits appropriate “status” labels and assigns appropriate “cause” labels to the issues. At the end of each data cycle, the DQ warehouse is automatically synchronized with the metadata on DQ issues from GitHub.

### Software Architecture

The DQ workflow was implemented using a combination of R, Python, and Go programming languages. A majority of the codebase uses R as it is specifically designed for statistical computing and data analysis. It is used to read data from a database and apply all DQ checks to said data. This may require manipulation and examination of large amounts of data at a time, e.g. in PEDSnet, a site may need to execute the DQ checks against 25 million records at a time in a given table. This is something that R makes simple and efficient using big data packages such as dplyr. Go is used to take the results of the DQ checks generated by the R code to create and post corresponding GitHub issues, as Go is a more general-purpose programming language, with a public API to interface with GitHub.

An ideal DQ workflow should be portable and capable of running on different systems, and R, Go, and Python languages offer this benefit in different ways. Since R is a scripting language, the scripts can be executed as long as the R binary is installed on the host machine. In the R portion of the source code, we use two ORM (object-relational mapping) libraries to help generate database queries that are cross-compatible with different RDBMS (relational database management system) such as PostgreSQL and Oracle. Go is a compiled programming language that can be compiled to target different machine types. Portability makes it easier to share DQ workflow source code and applications with different developers and data scientists to receive feedback. Python is used as a scripting language for resolving and updating new data quality results based on previous issues. As an interpreted and object-oriented programming language, Python makes it straightforward to encode and execute new conflict resolution modules (for investigation of difference between consecutive cycles) as necessary.

## Results

The DQ workflow has been executed in 13 data cycles over the course of the first two years (January 2015 – January 2017) of building PEDSnet. In this article, we evaluate and report the evolution of the workflow from different perspectives, including the underlying checks, issues reported to the sites, and the usage of the workflow by the sites. It should be noted that two of the eight partner sites in PEDSnet are virtual sites in that they do not send their datasets to the DCC and only participate remotely. For those sites, the first step of the workflow (apply DQ checks) is executed locally and the results are shared with the DCC for subsequent steps of the workflow. The in-house datasets are stored in a Postgres database, and the remote datasets are stored in Oracle databases. The average duration of executing the workflow in the most recent data cycle is 30 minutes/site.

### DQ Workflow Design

In general, the number of checks increases with each data cycle with an exception being the 10^th^ data cycle having a slight decrease due to a rigorous code review that removed some redundant checks from the workflow. The most recent cycle comprises 685 data quality checks drawn from 30 check types. In terms of the harmonized DQ terminology terms [8], the check catalog represents 29.34 percent completeness checks, 11.38 percent temporal plausibility checks, 28.9 percent atemporal plausibility checks, 29.78 percent value conformance checks, and 0.59 percent relational conformance checks.

It should be noted that the DQ checks do not include integrity constraint checks such as mandatory field checks, referential integrity checks, and unique key checks. Those constraints are validated prior to the execution of the workflow. In general, the DQ checks are designed for all CDM variables except a few, with the type of assessments being determined based on the type and importance of variable. In the most recent cycle, there are 66 fields with only one DQ check. The examples of such fields include (i) optional foreign keys, e.g. visit_occurrence.provider_id, where the *MissData* check is implemented, (ii) fields where the *InvalidValue* check is implemented, e.g. location.state, person.month_of_birth, visit_payer.plan_class, (iii) source value fields such as condition_occurrence.condition_source_value, person.gender_source_value where the *InconSource* check is implemented. There are 47 fields with two DQ checks, e.g. the numerical fields such as person.n_gestational_age have two checks: *MissData*, and *NumOutlier*. The most informative fields (total 52) have three or more checks, e.g. concept identifier fields where the *InvalidVocab, MissData, MissConID, MissFact, InconSource, InvalidMap* checks are implemented, and date fields where the *MissData, ImplPastDate, ImplFutureDate, ImplEvent, PreBirth, PostDeath, InconDateTime* checks are implemented. In addition, there are 18 table level checks where a table or multiple tables are analyzed without focusing on a certain field such as *InconCohort, MissVisitFact*, and *UnexDiff*. Furthermore, there are 24 fields in the CDM for which no DQ check was applicable, e.g. primary keys (person.person_id), mandatory foreign keys (person.care_site_id, measurement_organism.person_id), fields that are not transmitted to the DCC (e.g. NPI, DEA in Provider), and unstructured fields such as measurement.value_source_value and drug_exposure.sig.

### DQ Workflow Results

**Figure 2:**
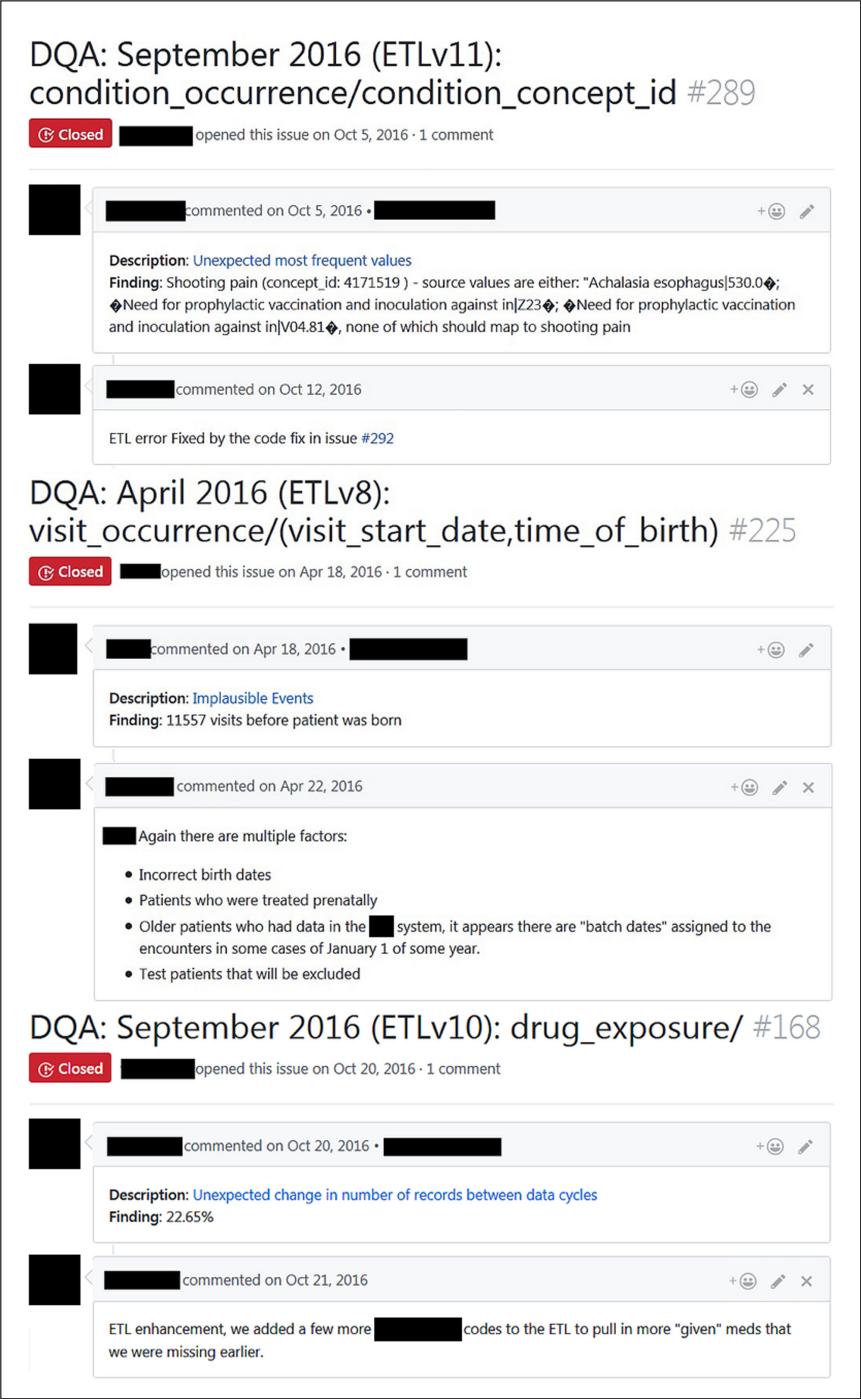
Examples of data quality issues posted on GitHub; sensitive data are hidden to preserve anonymity.

Figure 2 shows a screenshot of three PEDSnet DQ issues reported on GitHub; their backend metadata is illustrated in Table 1. Each GitHub issue describes the key information about the issue including the source fields in the header, and the executed check type with a hyperlink to the public source code that resulted into the issues and the findings in the body of the issue. More importantly, the GitHub issue provides a user-friendly collaborative space to discuss, track, and resolve (or find closures to) specific issues. In terms of the number of comments on GitHub issues, the average value is 1.56, mode and median are 1, and the range is 0 to 9. During the 13 cycles, the data developers used the system to identify 855 issues as characteristic issues, and resolve 1,483 ETL-based programming errors, of which 807 were due to ambiguities in network conventions.

**Figure 3:**
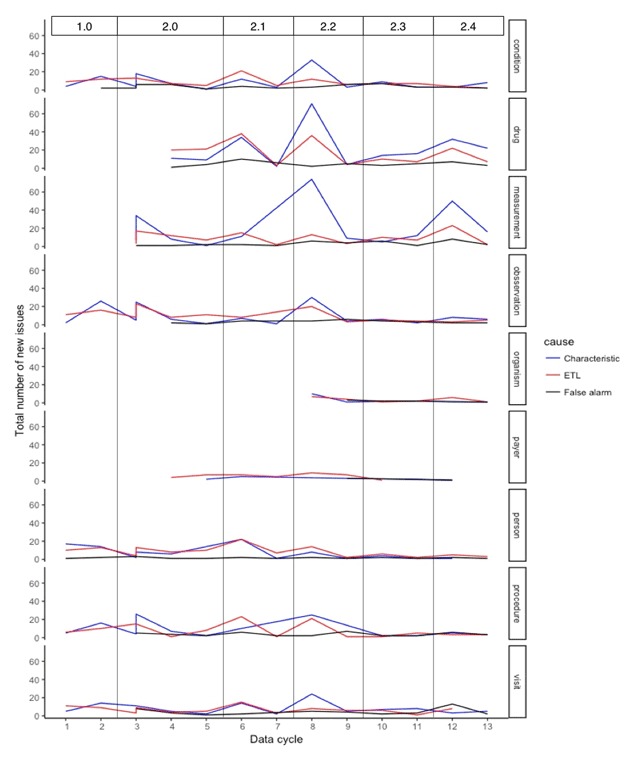
The domain-wise longitudinal distribution of number and types of reported issues. The top horizontal bar indicates the version number of network conventions adopted for a given data cycle.

Figure 3 shows the longitudinal domain-wise distribution of issues reported across data. The ETL issues represent the cases when the sites have spotted errors in the ETL code, i.e. programming errors, or errors due to ambiguity in the ETL conventions document. For all domains, the peaks in the number of ETL issues are due to the change in the network conventions for a given domain and represent associated changes in the ETL code for the affected domain. The characteristic issues represent a variety of cases that are unresolvable by the site due to incomplete source data capture, data entry errors, true anomalies, EHR configurations, administrative workflows, etc. The peaks, yet again, represent changes in the conventions document: addition of new fields or domains and hence learning of new data characteristics. The taller peaks represent the large number of columns for which the conventions have changed. The false alarms represent either a bug in the DQ workflow or an improvement in the site’s ETL process since the previous cycle. Since the DQ checks are continuously evolving, the changes in the codebase often lead to programming errors causing false alarms although in no identifiable pattern. In addition, some issues are identified as a result of natural expansion of datasets across data cycles (e.g. improvement in data capture).

**Figure 4:**
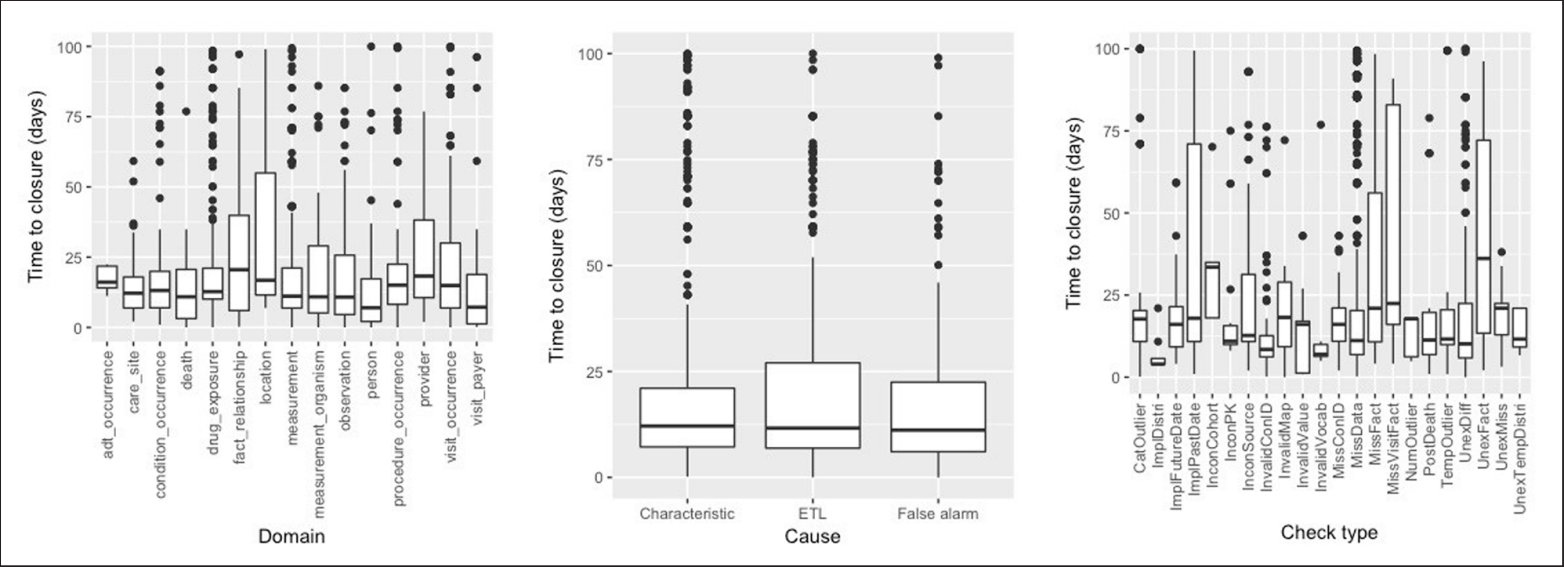
Distribution of GitHub issue closure duration across data domains, issue causes, and DQ check types.

Figure 4 displays the distribution of issue resolution duration measured against several entities. The y-axis denotes the time-to-closure (in days) of GitHub issues. This analysis was limited to the closed issues in GitHub, and included the data from six most recent data cycles. The median and interquartile range for site-wise issue duration was 14.81 days and 8.38 days, respectively. In addition, a difference in duration of submission (when a new convention is introduced, e.g. cycle 12) and resubmission (when the same convention is continued, e.g. cycle 13) was observed, e.g. the median and interquartile range for both cycles were 15.75 days (2.62 inter-quartile range), and 58.94 days (19.14 inter-quartile range), respectively. The domains with greatest variability include the auxiliary tables, fact_relationship, location, provider, and measurement_organism, where certain sites considered the associated issues to be lower priority and hence did not process them as quickly as other sites. The visit_occurrence and observation domains also have greater variability because of constantly changing conventions in these domains, and variability could be attributed to the field with evolving or unclear conventions versus the fields with straightforward resolutions. In terms of causes, while the median is about the same for different types of causes, the ETL issues have slightly higher variability reflecting the wide range of potential programming errors across issues. The check types *ImplPastDate, MissFact, MissVisitFact*, and *UnexFact*, have greater variability across issues and take longer to resolve, as these check types involve discussion with domain experts, accessing other local units to extract missing data, etc. The longer resubmission cycles are due to the ETL analysts having to split time between investigating the DQ issues and preparing data for the upcoming (new) conventions. It should be noted that the duration is only an approximation of issue resolution timeline and the closure of an issue on GitHub relies on many other factors such as staffing issues, local practices of handling and processing GitHub issues.

## Discussion

We have designed an end-to-end workflow to manage DQ assessments in a CDRN. The workflow has been developed iteratively over a period of two years using design suggestions from a variety of data users [18]. The system has been successfully implemented and is being routinely used by the PEDSnet CDRN for improving and quantifying the network data quality [19]. In a previous study [19], we provided a summary of the content of the PEDSnet data quality warehouse, highlighting missing data and outliers as most frequent issue types, and medications and lab measurements are most complex domains to ETL. In this study, we report the operational results of using the proposed DQ workflow in PEDSnet.

The continued increase in the number of checks across data cycles indicates that the workflow is being improved based on the lessons learned from the previous data cycles. The analysis indicates that new checks related to temporal plausibility, validation, and relational and computational conformance should be designed. The main source of evolution is due to engagement in science queries. The key manual intervention challenges in designing new checks include determination of the combination of fields and determination of thresholds for DQ checks. Also, since PEDSnet relies on using standard vocabularies for specific domains to support standardized queries, sites may be required to either use standard crosswalks, or manually derive and maintain some ETL mappings from source to standard PEDSnet vocabularies. Such manually maintained crosswalks (for domains like labs, organisms, specialty, route, race, etc.) are not only time intensive and error-prone, but also difficult to review from a DQ stand point leading to some undetected DQ problems. Also, the reliance on automatic crosswalks (e.g. OMOP’s ICD to SNOMED crosswalk) may lead to incorrect mappings due to changing nature of deprecated mappings and concepts in such crosswalks. In addition, methods like gold standard and log review [5] are not yet used in implementing the checks and will be considered in the future.

There are a few noteworthy systems designed for conducting data quality assessments on clinical data networks. The Sentinel Operations Center of the Sentinel Initiative [11,12,20] prepares the quality review and characterization package organized into multiple levels of DQ checks: compliance with the Sentinel CDM to ensure completeness and validity, cross-variable and cross-tabular accuracy and integrity, cross-ETL consistency of trends, and cross-ETL plausibility [21]. OHDSI’s Achilles [22] is another prominent and widely used general-purpose data quality assessment and characterization tool for the OMOP CDM. Also, the PCORI data characterization query package is made available to all data partners, and includes a range of data model conformance, data completeness, and data plausibility checks for the PCORI’s CDM [10]. Recently, Callahan et al. [9] highlighted the distribution of different types of assessments across six data sharing networks, including PEDSnet, and categorize the works into various maturity levels. The PEDSnet data quality program was considered equivalent to Sentinel, Kaiser Permanente’s Center for Effectiveness and Safety Research (CESR) [23], and PHIS [24], in terms of maturity level of the process. In PEDSnet, in addition to executing the proposed workflow, we also execute Achilles and PCORI data characterization program periodically. The checks included in the PEDSnet DQ workflow subsume the error detection tests incorporated in Achilles Heel, the error detection module of Achilles, and complement PCORI data checks. Thereby, after the thirteenth data cycle in PEDSnet, we have catalogued nearly 147 average issues per site, whereas Achilles Heel averages 22 errors per site and PCORI data characterization averages 4 exceptions per site. The proposed system is similar to existing systems in that we incorporate a variety of “crowdsourced” data quality checks, and differs from existing systems in that we provide an iterative workflow that conducts automated longitudinal analysis of not only data but also issues stored in a data quality warehouse, and also integrates an effective mechanism to communicate and manage specific issues with the data partners.

The workflow is publicly available (https://github.com/PEDSnet/Data-Quality-Analysis) for use against any network databases modeled using the OMOP CDM. To initiate the workflow, the user is required to set up a configuration file with database properties. Besides the PEDSnet DCC, ETL analysts at several sites of PEDSnet, and analysts at the SHOnet CDRN have also executed the workflow to improve their datasets. In PEDSnet, at the end of two years, the program has continued to identify new issues, and adjudicate old issues across the network, demonstrating the continued utility of the program in building and maintaining a large-scale network. The domain-wise analysis shows that once a new convention is introduced or an existing convention is altered, it takes the sites a couple of data cycles to successfully adopt the convention, while resolving ETL errors, and validating the associated characteristic issues. These findings provide reality check on the estimated duration of development and quality assurance of multi-institutional networks.

The GitHub based tracking system has received positive feedback from all users because of accessibility, documentation, discussion, and assistance in replication. Nevertheless, the sites spend a nearly equal and significant amount in investigation of all types of issues (ETL, characteristic, and false alarms), underlining the extent of human intervention involved in the execution of a DQ data cycle. While the investigation of ETL issues is worth the time of data developers and helps in improving the consistency of network data, characteristics issues are more useful towards study investigators and do not necessitate any change to the data or the underlying ETL programs, and the false alarms should be completely ignored by the users. To this end, we recently introduced a ranking system to assist the sites in prioritization of their workload by predicting the cause of the issues [25].

While the purpose of the DQ workflow is to improve or optimize data quality for research, the outcome of the workflow is largely geared towards identifying a wide variety of granular data problems and assisting data developers in resolving ETL errors and iteratively improving the network data. The bigger purpose of any DQ program is to assess that the research readiness of the overall network, given the validated data characteristic issues. The PEDSnet data has been successfully used in executing many scientific studies over the past couple of years. However, the development of an intelligent system to quantify research suitability of PEDSnet deserves much deeper discussion and exploration, beyond the scope of this manuscript. Nevertheless, PEDSnet has implemented several measures toward this, in addition to the series of data plausibility checks embedded in the DQ workflow. First, we recently added a layer of fitness validation to select a winner submission between successive data submissions, based on the data quality of elements considered important for health services and comparative effectiveness research, such as drug concepts, drug dose, frequency, lab codes and results, provider specialty, etc. Second, PEDSnet routinely participates in the PCORnet data characterization cycles, and uses the identified exceptions to inform the PEDSnet DQ program as well as the affected sites, to further improve the research suitability of network data. Finally, it should be noted that the readiness of a network ultimately would depend on the research question being asked. In practice, a PEDSnet data scientist is paired with a research study and works closely with the study team to determine the feasibility of the study based on data quality results from the workflow. In a previous publication [19], we have provided some recommendations on cross-linking study variables (covariates, outcomes, confounding) with DQ results using visual heat maps, to assist researchers in making the final judgment.

## Conclusions

We demonstrate here a publicly available iterative workflow for conducting DQ assessments on a CDRN. We have formalized the problem of DQ assessments in CDRN, and proposed a modular workflow allowing to extend to more check types, and model changes. The workflow is reproducible and could be executed on any OMOP-based CDRN. The proposed system, while overlapping with existing data quality tools, has unique automated features of cross-cycle difference investigation and GitHub-based issue tracking. While the system is largely designed for data developers, our key future work includes a supplementary tool that assists data consumers in searching, reviewing, and understanding the results of the DQ workflow.

## Additional Files

The additional files for this article can be found as follows:

10.5334/egems.294.s1 Figure S1. The conceptual schema for conducting data quality assessments in PEDSnet; for further information, refer to the online appendix in [10]. This figure is a reproduction of Figure 1 in [10]; permissions were obtained from the Oxford University Press and Copyright Clearance Center.

10.5334/egems.294.s2 Figure S2. Investigate difference and resolve conflicts between issues from consecutive data cycles.
