# Supplementary material for: Design and Refinement of a Data Quality Assessment Workflow for a Large Pediatric Research Network
Source: EGEMS (Wash DC). 2019 Aug 1;7(1):36. doi: 10.5334/egems.294 (PMC6676917; doi:10.5334/egems.294)
Supplement: Figure S1. — The conceptual schema for conducting data quality assessments in PEDSnet; for further information, refer to the online appendix in [10]. This figure is a reproduction of Figure 1 in [10]; permissions were obtained from the Oxford University Press and Copyright Clearance Center. [file egems-7-1-294-s1.pdf]

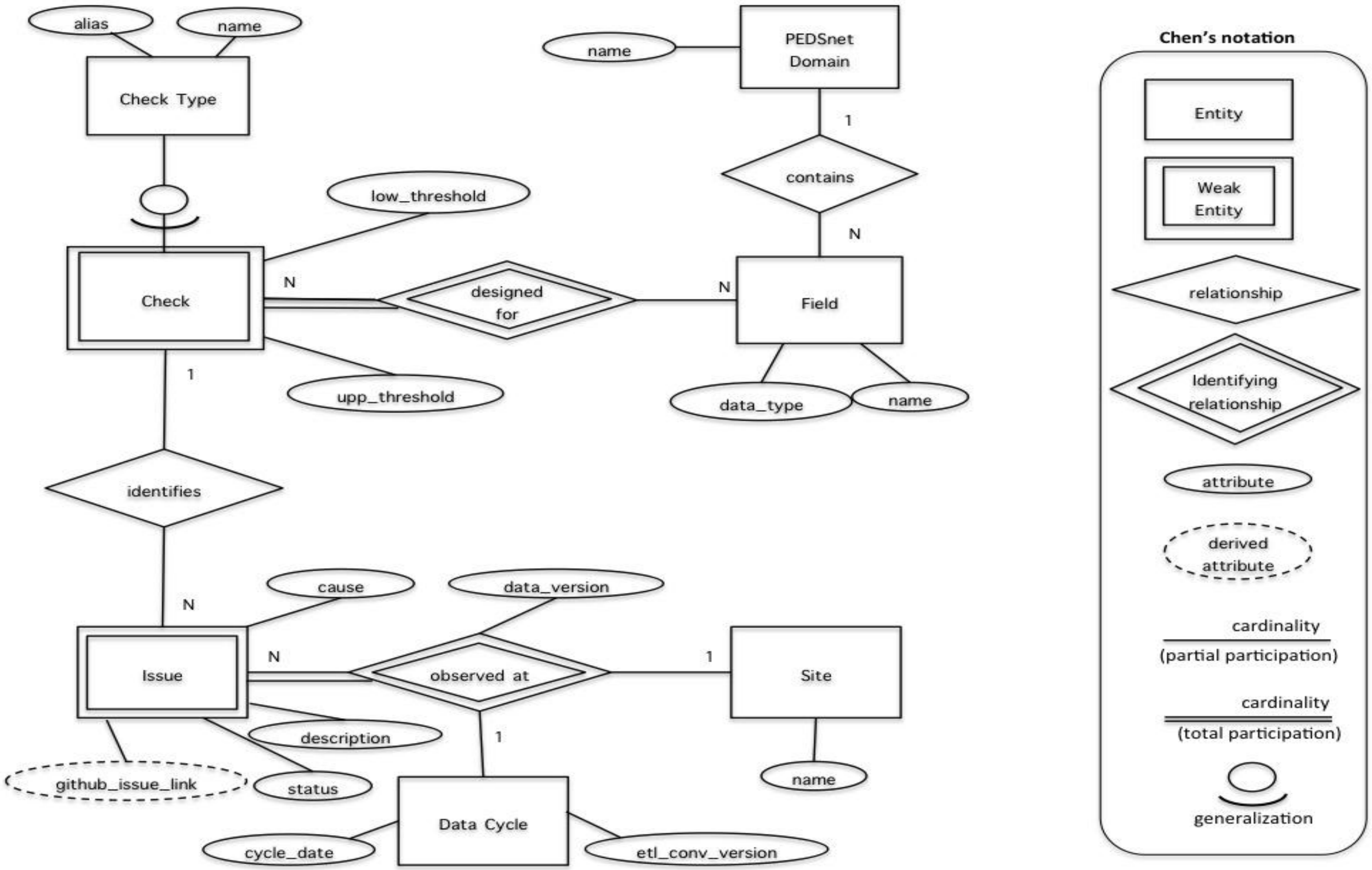

Figure S1. The conceptual schema for conducting data quality assessments in PEDSnet; for further information, refer to the online appendix in [10]. This figure is a reproduction of Figure 1 in [10]; permissions were obtained from the Oxford University Press and Copyright Clearance Center
