# Supplementary material for: Design and Refinement of a Data Quality Assessment Workflow for a Large Pediatric Research Network
Source: EGEMS (Wash DC). 2019 Aug 1;7(1):36. doi: 10.5334/egems.294 (PMC6676917; doi:10.5334/egems.294)
Supplement: Figure S2. — Investigate difference and resolve conflicts between issues from consecutive data cycles. [file egems-7-1-294-s2.pdf]

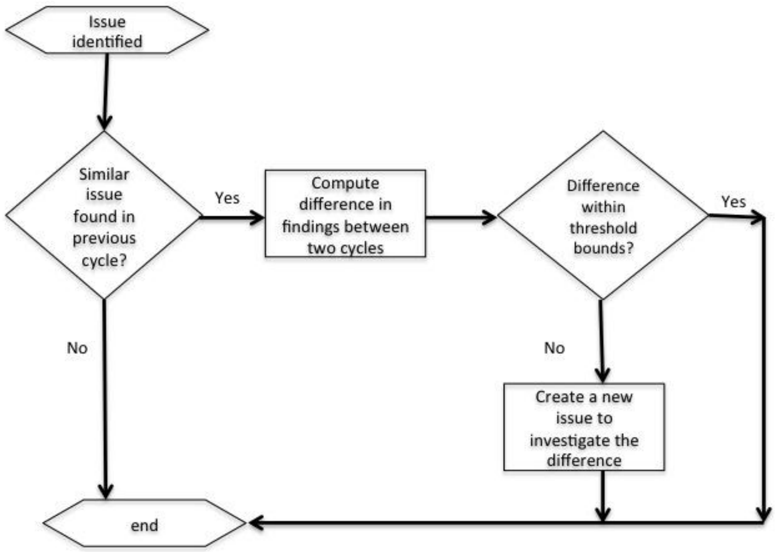

Figure S2. Investigate difference and resolve conflicts between issues from consecutive data cycles
